# A parent-report measure of children’s anxiety: psychometric properties of the Macquarie Anxiety Behavioural Scale (MABS) in a Chinese sample of preschool children

**DOI:** 10.1186/s40359-023-01463-1

**Published:** 2023-11-27

**Authors:** Wei Chen, Xingrong Zhou, Xingyu Yin, Shouying Zhao

**Affiliations:** 1 School of Psychology, Guizhou Normal University, Guiyang, China; 2 Center for Big Data Research in Psychology, Guizhou Normal University, Guiyang, China; 3 Bishan Gaoxin Middle School, Chongqing, China; 4 Kali University, Kali, China

**Keywords:** Macquarie Anxiety Behavioural Scale (MABS), Anxiety disorder, Anxiety behaviour, Preschool children, Measurement invariance

## Abstract

**Objective:**

The Macquarie Anxiety Behavioural Scale (MABS) is a newly developed scale to assess anxiety in children and teenagers. The present study aimed to evaluate the reliability and validity of the Chinese version of the MABS, as well as the measurement invariance across different age groups in a preschool-aged sample.

**Methods:**

A total of 1007 parents with children aged 3–6 years participated in the study. Internal consistency was assessed by calculating Cronbach’s alpha, McDonald’s omega and average inter-item correlation values. Confirmatory factor analysis (CFA) was conducted to examine the five-factor model. Multi-group CFA was conducted to test the measurement equivalence across different age groups (3- and 4-year-olds and 5- and 6-year-olds). Convergent, divergent, and criterion-related validity were assessed with Pearson correlation coefficients.

**Results:**

Internal consistency for the MABS total score was good and that of the subscales was acceptable. The CFA results showed that the five-factor structure of the MABS was supported in preschoolers (e.g., CFI = 0.929, TLI = 0.914, RMSEA = 0.050). In addition, scalar invariance of the MABS was supported across different age groups (e.g., ΔCFI = − 0.003, ΔTLI = 0, ΔRMSEA = 0). Furthermore, the MABS showed good convergent and divergent validity as well as criterion-related validity.

**Conclusion:**

The Chinese version of the MABS demonstrated satisfactory psychometric properties and appeared to be a valid and reliable instrument for measuring anxiety in preschool children.

## Introduction

Anxiety is a common emotional experience in children and adolescents [1], whereas those who are often in a state of excessive anxiety would be at higher risk of developing anxiety disorders. Although most research on anxiety in children and adolescents has focused on older children or adolescents, in recent years there has been a growing interest in the study of anxiety in preschool children [2]. Studies have found anxiety disorders are one of the most common mental health problems in pre-schoolers [3] and the prevalence of anxiety disorders in preschool children ranges from approximately 9.4% to even 22.2% [2–5]. Preschool children with anxiety disorders show considerable impairments [3]. Compared to preschoolers without anxiety disorders, those with anxiety disorders may have disrupted daily functioning [6] and thus are more likely to show lower positive affect and higher temperamental behavior inhibition [2, 7]. In addition, studies have found that anxiety problems in children tend to become chronic if the symptomatology starts early [8] and may have long-term adverse effects on adjustment [9, 10]. If not intervened, the presence of anxiety disorders in childhood may predict poor social skills, peer rejection, a lower sense of personal control [6], and poor academic performance later [11]. Even worse it may increase an individual’s risk of developing anxiety disorders [12–15] as well as various other psychiatric disorders, such as depression and conduct disorder later in life [1, 2, 15–18]. The impact of anxiety on young children is extensive; therefore, early detection and intervention of anxiety symptoms in preschool children are important and can provide window for shaping healthy social, emotional, and cognitive functioning through the lifespan [19].

Due to preschool children do not have the ability to self-report, obtaining information about young children’s anxiety symptoms by administrating parent-report rating scales is a relatively common method used in research. This is not only a time- and cost-effective method, but also can be helpful in the detection (i.e., screening) of young anxious children [20]. As far as we know, only the Preschool Anxiety Scale (PAS) [21] and the revised version of the Preschool Anxiety Scale [22] have been developed specifically to measure anxiety symptoms in preschool children and have been widely used [23–27]. Yet, both scales were developed based on the classification of the fourth edition of the Diagnostic and Statistical Manual of Mental Disorders (DSM-IV) [28] and one of their limitations is that using them requires parents to perceive and estimate their child’s anxiety level unless their child clearly expresses their concerns [21, 22, 29]. Whereas preschool children cannot talk about their emotions with great fluency and clarity sometimes, parents may not always be aware of the severity and frequency of the anxiety symptoms their child is experiencing [30]. Besides, studies also have shown that parents’ own emotional distress can influence their perception of the level of their child’s anxiety level [30–32]. Therefore, the information collected from parent-report scales (e.g., the Preschool Anxiety Scale) may have some bias that can reduce the accuracy of the measure. However, it is worth noting that some studies in typically developing children have found that when anxiety symptoms are more observable, a stronger parent-child agreement on the degree of anxiety symptoms in children can be found [33, 34]. Thus, it is better to use measures with more observable items to assess anxiety in preschoolers.

The Macquarie Anxiety Behavioural Scale (MABS) is a newly developed parent-report scale that focuses on observable behavioral indicators of anxiety [29]. Parents were only required to report the occurrence of their child’s anxious behaviour as mentioned in the items, independent of the parent’s perception of their child’s emotions. Therefore using the MABS to measure a child’s anxiety may be more valid and accurate. In addition, it was also designed to measure the five anxiety dimensions specified in the more recently published DSM-V [35], including generalized anxiety, panic, separation anxiety, social anxiety and specific phobia. The original version of the MABS was developed in Australia and ultimately 18 items were considered suitable for assessing anxiety in children and adolescents, irrespective of presence or absence of Autism Spectrum Disorder (ASD) [29].

Despite the potential of the MABS to be a useful parent-report instrument for the measurement of preschoolers’ anxiety, it has not been formally verified in preschool-aged samples, though a small number of preschoolers were included in the initial study [29]. Moreover, while measurement invariance of the MABS across ASD and non-ASD populations was demonstrated in the original study, we still do not know the measurement invariance of the MABS across different age groups. Some studies have found significant differences in anxiety levels between younger and older preschoolers. Notably, some found older preschoolers had higher levels of anxiety than younger preschoolers [24], while others found the opposite result [21, 25]. And at the same time, some studies did not find such an age difference [2]. Thus, measurement invariance of the MABS in this aspect is important as well. If the MABS performs inconsistently across different age groups, it may lead to serious problems, including misestimation of group differences, inaccuracy of study estimates, unfairness and inequity between samples [36–38]. Lastly, MABS has not yet been administered in the Chinese context, therefore, it is necessary to verify the applicability and generalizability of this scale for use in China.

The aim of the current study was to examine the psychometric properties of the Chinese version of the MABS in a sample of preschool children. For this purpose, we evaluated the reliability and validity of the Chinese version of the MABS. Firstly, the internal consistency was evaluated. Secondly, the factor structure of the MABS was examined. We hypothesized the factor structure of the MABS would be consistent with the original scale, with a five-factor structure. Thirdly, the measurement invariance of the MABS across different age (3- and 4-year-olds and 5- and 6-year-olds) groups were evaluated. We hypothesized that the measurement (at least scalar) invariance of the MABS would be established across different age groups. Finally, we analyzed the convergent and divergent validity as well as criterion-related validity of the MABS. We hypothesized that the MABS scores would correlate with scores of questionnaires that had either been used in previous studies measuring children’s externalizing and internalizing symptoms [22, 23], or measured variables had been proved to be associated with children’s anxiety, such as parenting stress [39, 40]. Based on the above psychometric tests, we expected to find satisfactory reliability and validity of MABS in our study.

## Methods

### Translation procedure

Before the development of the Chinese version of the MABS, we contacted one of the developers of the original MABS by email and obtained permission for the use of the scale in China. The Chinese translation of the MABS was developed by two independent groups through a back-translation procedure. One group consisted of four masters and one PhD in psychology, the other was a psychology master who passed the Test for English Majors-Band 8 (TEM-8). The initial Chinese version of the MABS was translated independently by the five-person group, and then back-translated to English by the other group. Then, we compared the inconsistencies between the back translation version and the original English version. Later we revised the inappropriate content in repeated iterations until the version was semantically identical and agreed both groups. The final version of the Chinese MABS was obtained through back-translation and discussion between the two groups.

### Procedure and participants

This research was approved by the committee of the School of Psychology, Guizhou Normal University. Participants were recruited from six public preschools in the city of Guiyang, located in Guizhou Province, Southwestern China. The researchers presented this study to the principals of six kindergartens based on personal contacts or collaborations in previous studies. All school principals agreed to participate in this study. Then teachers at these kindergartens sent a document explaining this research and an informed consent to the parents who might be potential participants. Only parents who provided a signed informed consent were invited to participate in the study. In addition, participants were informed that their answers would remain anonymous and were allowed to withdraw from this study at any time. The inclusion criteria for participants were: Chinese residency, fluency in Chinese, and being a parent of a child. Participants were excluded if their child was diagnosed with a developmental disorder (e.g., Autistic Disorder) or was currently undergoing psychological or psychiatric treatment. The questionnaires were finally delivered to 1046 parents with children aged 3–6 years old. However, 39 participants (3.7%) did not complete the survey, so they were excluded. The demographic characteristics of the sample were presented in Table 1. A total of 1007 parents (70.01% mothers) completed the survey. Their mean age was 33.9 years (SD, 5.0). Only 3.38% parents had a primary school education or below, 43.09% had a secondary school education or a high school education, and 53.53% had a university education or above.

**Table 1:** Descriptive information for the present sample (n = 1007)

Variables	Number (percentage)
Child age	
3 years old	123 (12.21%)
4 years old	352 (34.96%)
5 years old	340 (33.76%)
6 years old	192 (19.07%)
Parent	
Father	302 (29.99%)
Mother	705 (70.01%)
Educational level (mother/father)	
Primary education or below	34 (3.38%)
Middle school education	254 (25.22%)
High school education	180 (17.87%)
Undergraduate Education	474 (47.07%)
Postgraduate education or above	65 (6.45%)
Occupation (mother/father)	
staff of state organs or institutions	276 (27.41%)
employee of enterprises, companies or factories	219 (21.75%)
individual industrial and commercial households	95 (9.43%)
agricultural workers	56 (5.56%)
unemployees	103 (10.23%)
others	258 (25.62%)

### Measures

#### The Chinese version of the Macquarie Anxiety Behavioural Scale (MABS)

The MABS is a parent-report measure developed to assess anxiety in children and adolescents [29]. Following the recommendations of the original study [29], we translated the 18 items with good psychometrics performance into a Chinese version of the MABS. It consists of five subscales: generalized anxiety (4 items; e.g., “My child asked many questions about new situations”), panic (4 items; e.g., “My child suddenly started sweating and/or was unable to breathe even though there was no clear reason”), separation anxiety (3 items; e.g., “My child was unable to sleep on his/her own”), social anxiety (3 items; e.g., “My child became distressed by or avoided reading aloud, speaking or participating in class or during assembly”), specific anxiety (4 items; e.g., “My child avoided one or more of the following situations – the dark, crowds, heights, storms or water”). Parents were requested to rate each item on a five-point scale (1 = “never”, 5 = “all of the time”). The scores for the total scale and each subscale can be calculated by summing the responses of the relevant items. The higher the child’s MABS scores, the higher their anxiety level.

#### The strengths and difficulties questionnaire–parent version (SDQ-P)

The SDQ–P is a parent-report instrument designed to assess general difficulties and prosocial behavior of children [41]. It consists of 25 items grouped into five subscales (prosocial behavior, behavior problems, emotional symptoms, hyperactivity/inattention, and peer relationship problems) and is rated on a Likert-type scale ranging from 0 (not true) to 2 (certainly true). Recent research supported merging the four subscales of the SDQ-P into two subscales to reflect externalizing (conduct problems and hyperactivity/inattention) and internalizing (emotional symptoms and peer relationship problems) difficulties [42, 43]. In the current sample, the internal consistency (α) for the internalizing and externalizing subscales was 0.69 and 0.64, respectively. And the SDQ-P was used as a reliable tool to establish convergent and divergent validity of the MABS.

### The parenting stress index-short Form-15 item (PSI-SF-15)

The PSI-SF-15 [44] is a 15-item with a 5-point Likert-type (1 = “strongly disagree”, 5 = “strongly agree”) measure to assess parenting stress perceived by the caregivers. It consists of three subscales: parental distress (PD), parent-children dysfunctional interaction (PCDI) and difficult child (DC). The Cronbach’s α in this study for PSI-SF-15 was 0.90, and the Cronbach’s α for PD, PCDI, and DC subscales were 0.79, 0.85 and 0.82, respectively. Based on several previous studies reporting a relation between preschoolers’ anxiety and parents’ parenting stress [39, 40], the PSI-SF-15 was implemented to examine the criterion-related validity of MABS.

### Data analysis

Descriptive statistics for all scales were conducted by Stata (version 15.1) software. In order to assess the internal reliability of the MABS, Cronbach’s alpha (α), McDonald’s omega (ω) and average inter-item correlations (AIC) were estimated. Cronbach’s α < 0.60 demonstrated insufficient consistency; 0.60–0.69 indicated marginal consistency; 0.70–0.79 indicated acceptable consistency; 0.80–0.89 demonstrated good consistency; ≥ 0.90 demonstrated excellent consistency [45]. And according to some previous research, a minimum value of 0.60 for both α and ω was considered adequate [46, 47]. AIC values should theoretically range between 0.15 and 0.50 [48].

To confirm the original five-factor structure model of the MABS in this studied group, Confirmatory Factor Analysis (CFA) was conducted using Mplus 8.3 software [49]. Since some values of the skewness and kurtosis of the data in the current study were outside the range of -1 to + 1 (see Table 2), we conducted a maximum Likelihood estimation with an adjusted chi-square (MLM), which is robust to non-normality [50]. The Comparative Fit Index (CFI), the Tucker-Lewis index (TLI) and the Root Mean Square Error of Approximation (RMSEA) were used to evaluate the degree of the model fit. Values of CFI and TLI greater than 0.90 indicate an acceptable fit; RMSEA value below 0.06 and SRMR value below 0.08 indicate a relatively good fit [51].

**Table 2:** Means, SDs, skewness, kurtosis, Cronbach’s α, McDonald’s ω, AIC and factor loadings of the MABS (n = 1007)

Item	M	SD	Skewness	Kurtosis	Loading	Internal consistency
Factor 1: GAD						α = 0.58 ω = 0.58 AIC = 0.23
m1. My child took more care than other children to avoid making mistakes or getting in trouble …	3.18	0.93	-0.10	3.01	0.50	
m2. My child asked many questions about new situations	3.90	0.92	-0.78	3.50	0.39	
m3. My child talked about the worst thing that might happen in a situation	2.92	1.00	0.10	2.43	0.44	
m4. My child spent more time or effort than was needed preparing for activities …	2.89	0.96	0.13	2.65	0.68	
Factor 2: PANIC						α = 0.84 ω = 0.85 AIC = 0.35
m5. My child told me s/he felt s/he was going crazy	1.61	0.80	1.53	5.56	0.63	
m6. My child told me s/he does not want to participate in certain activities …	1.82	0.91	1.16	4.04	0.66	
m7. My child suddenly started sweating and/or was unable to breathe …	1.48	0.68	1.67	6.67	0.87	
m8. My child told me that s/he suddenly felt numbness or a tingling sensation	1.51	0.76	1.86	7.07	0.89	
Factor 3: SAD						α = 0.57 ω = 0.59 AIC = 0.40
m9. My child was unable to sleep on his/her own	2.42	1.18	0.41	2.14	0.48	
m10. My child talked about something bad that might happen when we’re not together	1.98	0.97	0.94	3.44	0.73	
m11. My child checked where I would be before separating or after …	3.06	1.23	-0.20	2.03	0.48	
Factor 4: SOC						α = 0.76 ω = 0.77 AIC = 0.51
m12. My child either became distressed by or avoided performing …	2.02	0.94	0.94	3.78	0.76	
m13. My child avoided talking to other people despite talking easily at home …	2.34	1.10	0.53	2.53	0.62	
m14. My child became distressed by or avoided reading aloud, speaking or participating in class or during assembly	2.13	0.95	0.80	3.40	0.80	
Factor 5: SPEC						α = 0.62 ω = 0.63 AIC = 0.36
m15. My child refused to travel in certain means of transport …	1.61	0.81	1.67	6.53	0.67	
m16. My child refused to be around certain animals or insects …	2.51	1.23	0.36	2.05	0.54	
m17. My child avoided one or more of the following situations – the dark, crowds, heights, storms or water	2.94	1.19	-0.13	2.04	0.45	
m18. My child refused one or more of the following - going to the doctor, going to the dentist, getting an injection	2.91	1.17	0.01	2.14	0.40	
Total	43.22	8.96	0.47	4.24		α = 0.82 ω = 0.83 AIC = 0.20

Note: AIC = average inter-item correlation; MABS = Macquarie Anxiety Behavioural Scale; GAD = Generalized anxiety disorder; SAD = Separation anxiety; SOC = Social anxiety; SPEC = Specific phobia

To ensure that the differences in the MABS scores were due to true observed individual differences in the measuring scale, and not to the measurement artifacts, a CFA-based technique was used to examine the measurement invariance across different age groups [52]. We divided the age group into 3–4 years (younger age group) and 5–6 years (older age group) to measure the invariance in the current study, as previous studies indicated that children’s anxiety symptoms vary significantly between these groups [25, 53]. Following previous research [54–56], several established procedures were performed to assess the measurement invariance of the MABS. First, configural invariance was evaluated to confirm the factor equivalence between groups. In the configural equivalence analysis, all parameters of the observed variables were estimated freely. Next, based on the results, metric invariance was then assessed to examine the equivalence of factor loading across groups. In the analysis of metric invariance, the factor loadings were restricted to be equivalent for the tested groups to determine whether items represent the same concept across groups. Metric invariance was considered to exist when there was no difference in the fit of the metric and configural models. Third, scalar invariance was assessed by constraining the item thresholds to be equal across groups. Scalar invariance was considered to exist when there was no difference in the fit of the scalar and metric models. Fourth, strict invariance was assessed to determine whether the error variances were equal across groups. Strict invariance was considered to establish when there was no difference in the fit of the strict and scalar models. According to previous recommendations [57, 58], ΔCFI < 0.010, ΔTLI < 0.010, and ΔRMSEA < 0.015 suggested a presence of invariance. Additionally, the independent sample t-test would be used to test age differences on the MABS scores only when measurement invariance was established. The mean difference in MABS total scores and all subscales scores between groups were compared through the independent samples t-test. The index of effect size was calculated by Cohen’s d [59].

Pearson correlation coefficients were calculated to assess the convergent and divergent as well as the criterion-related validity of the MABS. According to the some previous guidelines [59]: r between 0.10 and 0.30, small correlation; r between 0.30 and 0.50, medium correlation; and r > 0.50, strong correlation. The significance differences between the correlation coefficients were calculated using an online tool, the “cocor” [60], and following the previous recommendations [61].

## Results

### Descriptive statistics

#### Reliability

The Cronbach’s αs and McDonald’s ωs for the whole MABS scale (α = 0.82/ ω = 0.83), the Panic subscale (α = 0.84/ ω = 0.85) and the Social anxiety subscale (α = 0.76/ ω = 0.77) indicated good internal consistency. Internal consistency for the Specific anxiety subscale (α = 0.62/ ω = 0.63) was acceptable and poor for the subscale Generalized anxiety (α = 0.58/ ω = 0.58) and Separation anxiety (α = 0.57/ ω = 0.59). AIC value for the total MABS was 0.20, and on the MABS subscales, AIC for Generalized anxiety was 0.23, for Panic was 0.35, for Separation anxiety was 0.40, for Social anxiety was 0.51 and for Specific anxiety was 0.36 (see Table 2).

### Structure validity

**Fig. 1:**
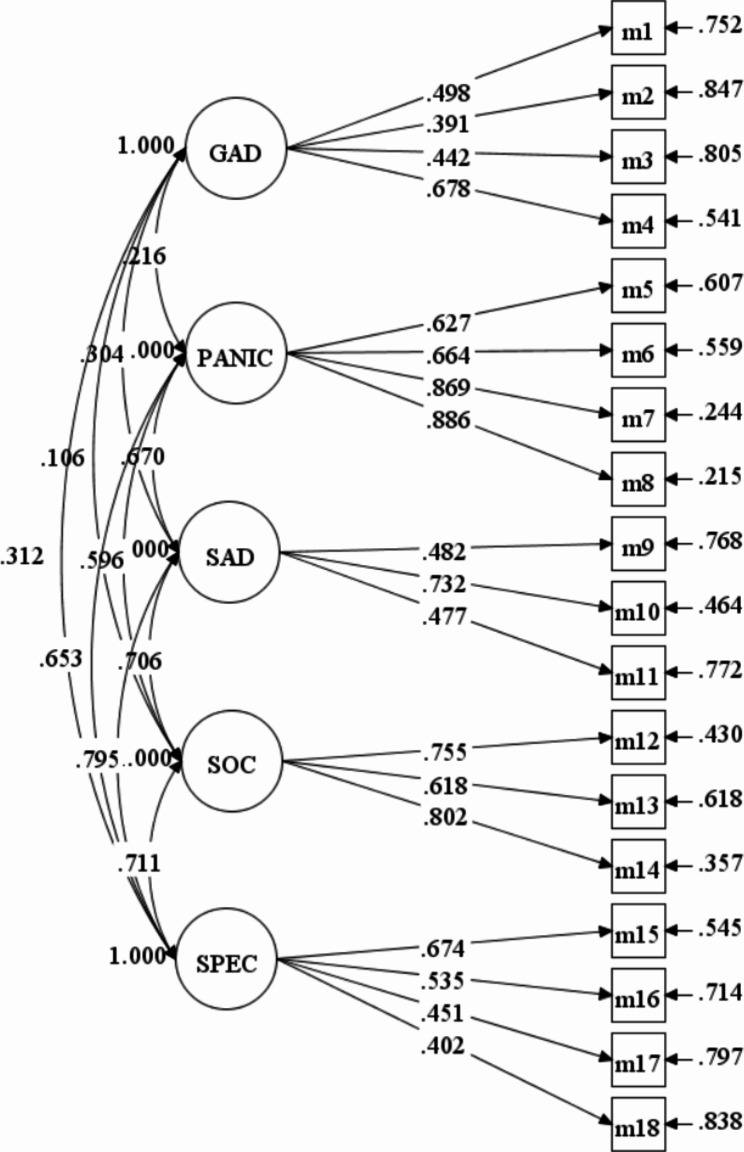
The five-factor model evaluated in the confirmatory factor analysis. GAD = Generalized anxiety disorder; SAD = Separation anxiety; SOC = Social anxiety; SPEC = Specific phobia

Since the MABS has shown a five-factor structure in the original study [29], which was also consistent with DSM-5, the current study directly examined the five-factor model of the MABS. The result showed a satisfactory fit for the five correlated factors based on DSM: CFI = 0.932, TLI = 0.917, RMSEA = 0.048, 90% CI [0.043 0.053], SRMR = 0.047. In the CFA model (see Fig. 1), items had moderate to high factor loadings on their underlying factor. Standardized factor loadings of Generalized anxiety ranged from 0.39 to 0.68, those of Panic from 0.63 to 0.89, those of Separation anxiety from 0.48 to 0.73, those of Social anxiety from 0.62 to 0.80, and those of Specific anxiety ranged from 0.40 to 0.67 (see Table 2).

### Measurement invariance

A multi-group confirmatory factor analysis (MCFA) was performed to examine the measurement invariance of the MABS across younger (3- and 4-year-olds) and older (5- and 6-year-olds) groups. Fit indices (CFI = 0.928, TLI = 0.912, RMSEA = 0.050) for the configural invariance model demonstrated that configural equivalence was established; therefore, this model was used as the baseline in subsequent testing. Satisfactory fit indices (ΔCFI = 0, ΔTLI = + 0.005, ΔRMSEA=-0.002) supported the metric invariance model. The scalar invariance model also yielded satisfactory fit indices (ΔCFI =-0.003, ΔTLI = 0, ΔRMSEA = 0). Fit indices for the strict invariance model were ΔCFI=-0.014, ΔTLI=-0.009, and ΔRMSEA = + 0.003 (see Table 3), not supporting the strict measurement invariance. Thus, these results support the hypothesis that the measurement invariance of the MABS, at least scalar invariance, were established across younger and older groups. Consequently, the independent-sample *t*-test was conducted, and total MABS scores were significantly higher among older group in comparison with the younger group (*t* = -4.49, *p* < 0.001, Cohen’s *d* = -0.28). Specifically, older group scored significantly higher on all subscales of the MABS compared to younger group (see Table 4).

**Table 3:** Model comparisons for measurement invariance testing across younger (3- and 4-year-olds: N = 475) and older (5- and 6-year-olds: N = 532) groups

Model	S-B χ^2^	df	CFI	TLI	RMSEA	ΔCFI	ΔTLI	ΔRMSEA	Decision
Model 1	563.758	250	0.928	0.912	0.050				
Model 2	574.305	263	0.928	0.917	0.048	0	0.005	-0.002	Accept
Model 3	600.235	276	0.925	0.917	0.048	-0.003	0	0	Accept
Model 4	678.980	294	0.911	0.908	0.051	-0.014	-0.009	0.003	Reject

NOTE: Model 1, configural invariance; Model 2, metric invariance; Model 3, scalar invariance; Model 4, residual invariance; S-B χ2, Satorra-Bentler scaled Chi-squared test; df, degrees of freedom; TLI, Tucker-Lewis index; CFI, comparative fit index; RMSEA, root-mean-square error of approximation; ΔCFI, ΔTLI, and ΔRMSEA values represent comparisons of in-row model with model immediately above the row

**Table 4:** Means, standard deviations (mean ± SD) and between sample differences for MABS (3- and 4-year-olds: N = 475; 5- and 6-year-olds: N = 532)

	3–4 years	5–6 years	*t*-value	Cohen’s *d*
GAD	12.63 ± 2.59	13.13 ± 2.45	-3.13**	-0.20
PANIC	6.12 ± 2.51	6.66 ± 2.64	-3.35***	-0.21
SAD	7.18 ± 2.43	7.71 ± 2.53	-3.38***	-0.21
SOC	6.28 ± 2.42	6.66 ± 2.49	-2.47*	-0.16
SPEC	9.68 ± 3.08	10.24 ± 3.00	-2.91**	-0.18
Total	41.89 ± 8.81	44.40 ± 8.92	-4.49***	-0.28

Note: GAD = Generalized anxiety disorder; SAD = Separation anxiety; SOC = Social anxiety; SPEC = Specific phobia

*, p < 0.05; **, p < 0.01; ***, p < 0.001

### Convergent, divergent validity and criterion-related validity

To further explore the convergent and divergent validity of the Chinese MABS, correlations between the MABS and SDQ-P subscales were calculated. As indicated in Table 5, the total score and subscales of the MABS correlated significantly with internalizing subscale of SDQ-P. Meanwhile, the total score and subscales of the MABS correlated significantly with externalizing subscale of SDQ-P, except with the GAD subscale. Z tests were conducted to determine whether the correlation between the MABS and internalizing subscale (convergent relationship) was significantly stronger than the correlation between MABS and externalizing subscale (divergent relationship). The correlation coefficients between the total scale and subscales and internalizing problems were significantly stronger than the ones between the total scale and subscales and externalizing subscale except that the correlation between the SAD and internalizing problems was not significantly stronger than the one between SAD and externalizing subscale.

**Table 5:** Correlations between the MABS and Internalizing subscale and Externalizing subscale of SDQ-P.

	Internalizing	Externalizing	Z
1. MABS	0.49**	0.31**	6.16**
2.GAD	0.07*	-0.05	
3.PANIC	0.44**	0.26**	5.98**
4.SAD	0.37**	0.32**	1.65
5.SOC	0.46**	0.30**	5.41**
6.SPEC	0.33**	0.22**	3.53**

NOTE: MABS = Macquarie Anxiety Behavioural Scale; GAD = Generalized anxiety disorder; SAD = Separation anxiety; SOC = Social anxiety; SPEC = Specific phobia

*, p < 0.05; **, p < 0.01

With regard to criterion-related validity, the results showed that, with the exception of the GAD subscale, there were moderate to strong correlations between the MABS and its subscales and PSI-SF-15 as well as its three subscales, ranging from 0.32 to 0.57 (*p* < 0.01) (see Table 6).

**Table 6:** Correlations between the MABS and PSI-SF-15.

	1	2	3	4	5	6	7	8	9	10
1. MABS	1									
2. GAD	0.46**	1								
3. PANIC	0.72**	0.15**	1							
4. SAD	0.74**	0.18**	0.44**	1						
5. SOC	0.72**	0.07*	0.49**	0.47**	1					
6. SPEC	0.75**	0.18**	0.38**	0.46**	0.44**	1				
7. PSI-SF-15	0.57**	0.05	0.46**	0.47**	0.53**	0.42**	1			
8. PD	0.47**	0.04	0.38**	0.39**	0.41**	0.37**	0.84**	1		
9. PCDI	0.52**	0.06	0.49**	0.38**	0.50**	0.35**	0.85**	0.62**	1	
10. DC	0.46**	0.03	0.32**	0.43**	0.43**	0.36**	0.84**	0.54**	0.56**	1

NOTE: MABS = Macquarie Anxiety Behavioural Scale; GAD = Generalized anxiety disorder; SAD = Separation anxiety; SOC = Social anxiety; SPEC = Specific phobia; IN = Internalizing subscale of SDQ-P; EX = Externalizing subscale of SDQ-P; PSI-SF-15 = the Parenting Stress Index-Short Form-15 item; PD = Parenting distress subscale of PSI-SF-15; PCDI = Parent–children dysfunctional interaction subscale of PSI-SF-15; DC = Difficult child subscale of PSI-SF-15.

*, p < 0.05; **, p < 0.01

## Discussion

The MABS is a newly developed parent-report scale for assessing anxiety in children and adolescents [29]. The current study examined the psychometric properties of the Chinese version of MABS, especially its measurement invariance across different age groups in a sample of preschool children. To our best knowledge, this was the first research to further explore the reliability and validity of the MABS. Our findings indicated that the Chinese version of the MABS had acceptable reliability and good validity, as well as measurement invariance in a preschool sample, suggesting that this instrument can be relied upon to measure anxiety in preschool children.

The MABS showed acceptable internal consistency (see Table 2). The Cronbach’s α of the whole MABS (α = 0.82) was lower compared to the original MABS (α = 0.97) [29], but still satisfactory. Although the reliability of the subscales was not mentioned in the original study, we further examined the reliability of its subscales. Three of these five subscales showed α and ω values above 0.60, while the Generalized anxiety subscale (α = 0.58/ ω = 0.58) and Separation anxiety subscale (α = 0.57/ ω = 0.59) had slightly lower scores. Therefore, we also calculated the average inter-item correlations (AIC), which was another way of analyzing internal consistency independent of the number of items and sample size. The results showed that AICs of Generalized anxiety and Separation anxiety subscales were within the recommended range, indicating both constructs of the MABS were formed with reasonably homogenous items. Overall, the MABS had acceptable reliability and was still a reliable scale.

In reference to the structure validity, the CFA result in our study yielded support for the correlated five-factor structure of the MABS in the preschool-aged sample. This result was consistent with the original research [29], which may indicate that the five-factor model of the MABS was stable in different cultures and can be used in both Western and Eastern countries. Although in the original study, the best model obtained by the researchers was five correlated DSM factors with correlated errors, which was because the original sample was a population with autism spectrum disorder (ASD) and the similarity and overlap between the items can be explained by the way anxiety presented in ASD [29]. In contrast, our study was not conducted in an ASD sample, where those similarities and overlaps between items may not exist, so the MABS obtained a more concise factor structure in this study. Overall, the above result demonstrated the satisfactory structural validity of the MABS in China.

The measurement invariance of a scale should be checked before comparing scale scores across groups [56, 62]. As a result, we examined the measurement invariance of the MABS across the younger (3- and 4-year-olds) and older (5- and 6-year-olds) groups, by assessing configural, metric, scalar and strict invariance. The result of the configural invariance assessment showed that the number of factors and factor patterns were equivalent in different age groups. The metric invariance assessment showed that the observed items and underlying factors of this instrument were equal in different age groups. The scalar invariance assessment indicated that cross-group differences in the means of the observed variables reflected inter-group differences in the means of the latent variables. While the strict invariance assessment showed that the error variance of each group was not equivalent in this study. In most previous empirical studies, researchers had argued the evaluation of strict invariance was too strict and unrealistic [63]. In short, our findings supported the measurement invariance of the MABS, at least for different age groups with strong invariance of anxiety measurement. This implied that the construct measured by the MABS has the same meaning across different age groups and it could provide accurate information for group comparisons.

Since we were able to confirm that factor loadings and intercepts were equivalent across younger and older groups in our measurement invariance analyses, we could conclude that older group (5- and 6-year-olds) have higher anxiety levels in comparison to younger group (3- and 4-year-olds) in this preschool sample. Similar to previous studies, older preschoolers had significantly higher levels of anxiety than younger preschoolers [24], which was also consistent with some earlier research that some anxiety problems increase as a function of cognitive development [64, 65].

The MABS showed good convergent and divergent validity, as in general the correlations between the MABS and internalizing subscale of SDQ-P were more strongly than the ones between MABS and externalizing subscale of SDQ-P. This indicated that child’s anxiety as measured by the MABS showed more internalizing rather than externalizing symptoms. It should be noted that the correlation between SAD and internalizing subscale was not significantly higher than the one with externalizing subscale. Nevertheless, the correlations between the two showed such a trend. With reference to the criterion-related validity, the correlations between the MABS and PSI-SF-15 indicated that the child’s anxiety levels were correlated with parenting pressures. Similar to previous studies, the child’s anxiety was related to parents’ parenting stress [39]. This may be due to the fact that children with anxiety symptoms often have obvious behavioral problems [25], and their parents may have to give more and experience more frustration [66], making parent-child interactions unpleasant and increasing the parenting stress of parents. It is notable that GAD did not show such a relationship in the present study, which may be due to the fact that children’s general anxiety behaviours do not cause a particularly high level of parenting stress from their parents, but this needs to continue to be verified in future studies.

Several limitations of this study should be noted. First, this study was not based on random sampling. We used a convenience sample of preschoolers from southwestern China. Thus, it remains unknown whether the current results could be generalized to other geographic areas in China. Future studies should replicate these findings in other regions of China. Secondly, due to limited sources, we failed to include a sample of clinical participants to explore the psychometric properties of the MABS among them. Some studies suggest that children with autism have higher levels of anxiety than normally developing children [67]. It is therefore necessary to validate the scale equally in a clinical sample, such as the measurement invariance of the MABS, to be sure that what was being compared was the same. Further studies that include clinical participants, such as ASD population, would be necessary.

Despite these limitations, the current study provided the first psychometric evaluation of the MABS in a large sample of Chinese preschool children. Further, it was the first to explore and verify the measurement invariance of the MABS across different age groups, which was considered a prerequisite for comparison among groups.

## Conclusion

In summary, the Chinese version of the MABS was demonstrated to have good psychometric properties in preschoolers and could be employed as a valid and reliable questionnaire to assess the possible anxiety symptoms in preschoolers. This effort broadened the psychometric properties of MABS and had important implications for empirical research in the prevention and treatment of anxiety in preschool children.

## Data Availability

The datasets used and/or analyzed during the current study are available anytime from the corresponding author on reasonable request.

## References

[1] Beesdo K, Knappe S, Pine D (2009). Anxiety and anxiety disorders in children and adolescents: developmental issues and implications for DSM-V. Psychiatr Clin North Am.

[2] Dougherty LR, Tolep MR, Bufferd SJ, Olino TM, Dyson M, Traditi J, Rose S, Carlson GA, Klein DN (2013). Preschool anxiety disorders: Comprehensive assessment of clinical, demographic, temperamental, familial, and life stress correlates. J Clin Child Adolesc Psychol.

[3] Egger HL, Angold A (2006). Common emotional and behavioral disorders in preschool children: presentation, nosology, and epidemiology. J Child Psychol Psychiatry.

[4] Bufferd SJ, Dougherty LR, Carlson GA, Klein DN (2010). Parent-reported mental health in preschoolers: findings using a diagnostic interview. Compr Psychiatr.

[5] Paulus FW, Backes A, Sander CS, Weber M, von Gontard A (2015). Anxiety disorders and behavioral inhibition in preschool children: a population-based study. Child Psychiatry Hum Dev.

[6] Rapee R, Kennedy S, Ingram M, Edwards S, Sweeney L (2005). Prevention and early intervention of anxiety disorders in inhibited preschool children. J Consult Clin Psychol.

[7] Lavigne JV, Hopkins J, Gouze KR, Bryant FB (2015). Bidirectional influences of anxiety and depression in young children. J Abnorm Child Psychol.

[8] Letcher P, Sanson A, Smart D, Toumbourou JW (2012). Precursors and correlates of anxiety trajectories from late childhood to late adolescence. J Clin Child Adolesc Psychol.

[9] Bodden DH, Dirksen CD, Bögels SM (2008). Societal burden of clinically anxious youth referred for treatment: a cost-of-illness study. J Abnorm Child Psychol.

[10] Merikangas KR, He JP, Burstein M, Swanson SA, Avenevoli S, Cui L, Benjet C, Georgiades K, Swendsen J (2010). Lifetime prevalence of mental disorders in U.S. adolescents: results from the National Comorbidity Survey replication–adolescent supplement (NCS-A). J Am Acad Child Adolesc Psychiatry.

[11] Essau CA, Conradt J, Petermann F (2000). Frequency, comorbidity, and psychosocial impairment of depressive disorders in adolescents. J Adolesc Res.

[12] Kim-Cohen J, Caspi A, Moffitt TE, Harrington H, Milne BJ, Poulton R (2003). Prior juvenile diagnoses in adults with mental disorder: developmental follow-back of a prospective-longitudinal cohort. Arch Gen Psychiatry.

[13] Woodward LJ, Fergusson DM (2001). Life course outcomes of young people with anxiety disorders in adolescence. J Am Acad Child Adolesc Psychiatry.

[14] Finsaas MC, Bufferd SJ, Dougherty LR, Carlson GA, Klein DN (2018). Preschool psychiatric disorders: Homotypic and heterotypic continuity through middle childhood and early adolescence. Psychol Med.

[15] Whalen DJ, Sylvester CM, Luby JL (2017). Depression and anxiety in preschoolers: a review of the past 7 years. Child Adolesc Psychiatr Clin N Am.

[16] Bittner A, Egger HL, Erkanli A, Costello EJ, Foley DL, Angold A (2007). What do childhood anxiety disorders predict?. J Child Psychol Psychiatry.

[17] Garber J, Weersing VR (2010). Comorbidity of anxiety and depression in youth: implications for treatment and prevention. Clin Psychol Sci Pract.

[18] Prinzie P, Harten L, Deković M, Akker A, Shiner R (2014). Developmental trajectories of anxious and depressive problems during the transition from childhood to adolescence: personality × parenting interactions. Dev Psychopathol.

[19] Zaim N, Harrison J (2020). Pre-school mental health disorders: a review. Int Rev Psychiatry.

[20] Olofsdotter S, Sonnby K, Vadlin S, Furmark T, Nilsson KW (2016). Assessing adolescent anxiety in general psychiatric care: diagnostic accuracy of the Swedish self-report and parent versions of the Spence Children’s anxiety scale. Assessment.

[21] Spence SH, Rapee R, McDonald C, Ingram M (2001). The structure of anxiety symptoms among preschoolers. Behav Res Ther.

[22] Edwards SL, Rapee RM, Kennedy SJ, Spence SH (2010). The assessment of anxiety symptoms in preschool-aged children: the revised preschool anxiety scale. J Clin Child Adolesc Psychol.

[23] Leung GSM, Yau KC, Yuen SY (2018). Validation of the Preschool anxiety scale-traditional Chinese (PAS-TC) in Hong Kong. Appl Res Qual Life.

[24] Broeren S, Muris P (2008). Psychometric evaluation of two new parent-rating scales for measuring anxiety symptoms in young Dutch children. J Affect Disord.

[25] Wang M, Zhao J (2015). Anxiety disorder symptoms in Chinese preschool children. Child Psychiatry Hum Dev.

[26] Ding X, Wang J, Li N, Su W, Wang H, Song Q, Guo X, Liang M, Qin Q, Sun L et al. Individual, prenatal, perinatal, and family factors for anxiety symptoms among preschool children. Front Psychiatry 2021, 12. 10.3389/fpsyt.2021.778291 PMC8721098 34987428

[27] Hudson JL, Murayama K, Meteyard L, Morris T, Dodd HF (2019). Early childhood predictors of anxiety in early adolescence. J Abnorm Child Psychol.

[28] American Psychiatric Association (1994). Diagnostic and statistical Manual of Mental disorders.

[29] Toscano R, Hudson JL, Baillie AJ, Lyneham HJ, McLellan LF (2020). Development of the Macquarie Anxiety Behavioural Scale (MABS): a parent measure to assess anxiety in children and adolescents including young people with autism spectrum disorder. J Anxiety Disord.

[30] Glod M, Creswell C, Waite P, Jamieson R, McConachie H, Don South M, Rodgers J (2017). Comparisons of the factor structure and measurement invariance of the Spence Children’s anxiety scale-parent version in children with autism spectrum disorder and typically developing anxious children. J Autism Dev Disord.

[31] Krain AL, Kendall PC (2000). The role of parental emotional distress in parent report of child anxiety. Clin Child Psychol Psychiatry.

[32] Niditch LA, Varela RE (2011). Mother–child disagreement in reports of child anxiety: effects of child age and maternal anxiety. J Anxiety Disord.

[33] Blakeley-Smith A, Reaven J, Ridge K, Hepburn S (2012). Parent–child agreement of anxiety symptoms in youth with autism spectrum disorders. Res Autism Spectr Disorders.

[34] Ooi YP, Weng SJ, Magiati I, Ang RP, Goh TJ, Fung DS, Sung M (2016). Factors influencing agreement between parent and child reports of anxiety symptoms among children with high-functioning autism spectrum disorders. J Dev Phys Disabil.

[35] American Psychiatric Association (2013). Diagnostic and statistical Manual of Mental disorders.

[36] Millsap RE, Yun-Tein J (2004). Assessing factorial invariance in ordered-categorical measures. Multivar Behav Res.

[37] Putnick DL, Bornstein MH (2016). Measurement invariance conventions and reporting: the state of the art and future directions for psychological research. Dev Rev.

[38] Vandenberg RJ, Lance CE (2000). A review and synthesis of the measurement invariance literature: suggestions, practices, and recommendations for organizational research. Organizational Res Methods.

[39] Wilkinson K, Ball S, Mitchell SB, Ukoumunne OC, O’Mahen HA, Tejerina-Arreal M, Hayes R, Berry V, Petrie I, Ford T (2021). The longitudinal relationship between child emotional disorder and parental mental health in the British child and Adolescent Mental Health surveys 1999 and 2004. J Anxiety Disord.

[40] Rodriguez CM (2011). Association between Independent reports of maternal parenting stress and children’s internalizing symptomatology. J Child Fam stud.

[41] Goodman R (1997). The strengths and difficulties questionnaire: a research note. J Child Psychol Psychiatry.

[42] Dickey WC, Blumberg SJ (2004). Revisiting the factor structure of the strengths and difficulties questionnaire: United States, 2001. J Am Acad Child Adolesc Psychiatry.

[43] Goodman A, Lamping DL, Ploubidis GB (2010). When to use broader internalising and externalising subscales instead of the hypothesised five subscales on the strengths and difficulties Questionnaire (SDQ): data from British parents, teachers and children. J Abnorm Child Psychol.

[44] Luo J, Wang MC, Gao Y, Zeng H, Yang W, Chen W, Zhao S, Qi S (2021). Refining the parenting stress index-short form (PSI-SF) in Chinese parents. Assessment.

[45] Barker C, Pistran N, Elliot R (1994). Research methods in clinical and counselling psychology.

[46] DeVellis RF (1991). Scale development: theory and applications.

[47] Oliden PE, Zumbo BD (2008). Coeficientes de fiabilidad para escalas de respuesta categórica ordenada. Psicothema.

[48] Clark LA, Watson D (1995). Constructing validity: basic issues in objective scale development. Psychol Assess.

[49] Muthén LK, Muthén BO. *Mplus user’s guide*. Eighth edn. Los Angeles, CA: Muthén & Muthén; 1998–2017.

[50] George D, Mallery M (2010). Using SPSS for Windows step by step: a simple guide and reference.

[51] Hu L, Bentler PM (1999). Cutoff criteria for fit indexes in covariance structure analysis: conventional criteria versus new alternatives. Struct Equ Model.

[52] Cheng C, Dong D, He J, Zhong X, Yao S (2020). Psychometric properties of the 10-item Connor-Davidson Resilience Scale (CD-RISC-10) in Chinese undergraduates and depressive patients. J Affect Disord.

[53] Hale WW, Crocetti E, Raaijmakers QAW, Meeus WHJ (2011). A meta-analysis of the cross-cultural psychometric properties of the screen for child anxiety related Emotional disorders (SCARED). J Child Psychol Psychiatry.

[54] Meredith W, Teresi JA (2006). An essay on measurement and factorial invariance. Med Care.

[55] Gregorich SE (2006). Do self-report instruments allow meaningful comparisons across diverse population groups? Testing measurement invariance using the confirmatory factor analysis framework. Med Care.

[56] Meredith W (1993). Measurement invariance, factor analysis and factorial invariance. Psychometrika.

[57] Cheung GW, Rensvold RB (2002). Evaluating goodness-of-fit indexes for testing measurement invariance. Struct Equation Model Multidisciplinary J.

[58] Chen FF (2007). Sensitivity of goodness of fit indexes to lack of measurement invariance. Struct Equation Modeling: Multidisciplinary J.

[59] Cohen J (1988). Statistical power analysis for the behavioral sciences.

[60] Diedenhofen B, Musch J (2015). Cocor: a comprehensive solution for the statistical comparison of correlations. PLoS ONE.

[61] Meng XL, Rosenthal R, Rubin DB (1992). Comparing correlated correlation coefficients. Psychol Bull.

[62] Han K, Colarelli SM, Weed NC (2019). Methodological and statistical advances in the consideration of cultural diversity in assessment: a critical review of group classification and measurement invariance testing. Psychol Assess.

[63] Van De Schoot R, Schmidt P, Beuckelaer A, Lek K, Zondervan-Zwijnenburg M (2015). Editorial: measurement invariance. Front Psychol.

[64] Muris P, Merckelbach H, Meesters C, van den Brand K (2002). Cognitive development and worry in normal children. Cogn Therapy Res.

[65] Westenberg PM, Drewes MJ, Goedhart AW, Siebelink BM, Treffers PD (2004). A developmental analysis of self-reported fears in late childhood through mid-adolescence: Social-evaluative fears on the rise?. J Child Psychol Psychiatry.

[66] Abidin R (1995). Parenting stress index, short form.

[67] MacNeil BM, Lopes VA, Minnes PM (2009). Anxiety in children and adolescents with Autism Spectrum disorders. Res Autism Spectr Disorders.

